# Training of Brazilian indigenous nurses: between human rights, valuing diversity and inclusion

**DOI:** 10.1590/0034-7167-2023-0430

**Published:** 2024-12-13

**Authors:** Nádile Juliane Costa de Castro, Ezequiel Sakew Wai Wai, Fernanda Teixeira Paes, Nyvia Cristina dos Santos Lima

**Affiliations:** I Universidade Federal do Pará. Belém, Pará, Brazil

**Keywords:** Nursing, Indigenous Peoples, Human Rights, Equity, Diversity, and Inclusion, Sustainable Development, Enfermeros y Enfermeras, Pueblos Indígenas, Derechos Humanos, Diversidad, Equidad e Inclusión, Desarrollo Sostenible

## Abstract

**Objectives:**

to analyze the possibilities and potential of training indigenous nurses, given the Brazilian Health System (SUS), understanding the relationships between education and health.

**Methods:**

theoretical-reflective study, based on scientific literature, aligned with the experience, critical thinking of its authors and the Sustainable Development Goals in Brazil.

**Results:**

this text articulates three axes: Potential for including indigenous students in nursing training; Paths to achieving equity through inclusion and retention policies for indigenous students at different levels; and Implications of this for the SUS and global health.

**Final Considerations:**

indigenous students, beneficiaries of affirmative actions, face challenges of inclusion and retention in public universities that directly impact their academic training. Added to this are the difficulties identified in basic education, professor training and implementation of permanence policies, with consequences for services and training at other levels.

## RESUMEN

**Objetivos:**

analizar las posibilidades y potencialidades de la formación de enfermeros indígenas, dado el Sistema Único de Salud (SUS), entendiendo las relaciones entre educación y salud.

**Métodos:**

estudio teórico-reflexivo, basado en literatura científica, alineado con la experiencia, el pensamiento crítico de sus autores y las metas de los Objetivos de Desarrollo Sostenible en Brasil.

**Resultados:**

este texto articula tres ejes: Potencial para la inclusión de estudiantes indígenas en la formación de enfermería; Caminos para alcanzar la equidad a través de políticas de inclusión y retención de estudiantes indígenas en los diferentes niveles; e Implicaciones de esto para el SUS y la salud global.

**Consideraciones Finales:**

los estudiantes indígenas, beneficiarios de acciones afirmativas, enfrentan desafíos de inclusión y retención en las universidades públicas que impactan directamente en su formación académica. A esto se suman las dificultades identificadas en la educación básica, la formación docente y la implementación de políticas de permanencia, con consecuencias para los servicios y la formación en otros niveles.

## INTRODUCTION

In Brazil, there are 1.6 million indigenous people with rights provided for in the 1988 Federal Constitution and in international treaties, including indigenous students, for whom access to higher education is a necessary right to reduce social and health inequities^(1)^. Training indigenous people as academics in health courses, which this study deals with, reaffirms the principle of equity, signaled in the Brazilian Health System (SUS - *Sistema Único de Saúde*) and in the SUS Indigenous Healthcare Subsystem (SASISUS - *Subsistema de Atenção à Saúde Indígena do SUS*), designed to base on the singularities of different indigenous peoples. The importance of the participation of indigenous people and their visions of health and well-being^(2,3)^ is recognized here, which are fundamental for dialoguing and confronting the inequities present in different health contexts^(1,2)^.

Studies in this sense bring to light debates about equitable processes and structures, dialoguing about the pluriethnic character of the Brazilian population^(3)^. They also deal with the potential impacts of public policies in Brazil, such as the Quota Law, formalized in Federal Law 12.711 of August 29, 2012, updated by Law 14.723/2023, which ensures that indigenous people have the opportunity to access higher education through citizenship and diversity perspectives, incorporated at all levels^(1,4)^.

This policy recognizes ethnic-racial diversity and protects indigenous peoples’ ways of being^(3,4)^, being important for the intercultural training of all healthcare professionals who participate in it, provoking new dynamics in universities, enabling dialogues and interactions between different cultures and production of knowledge, from interdisciplinary and diversity perspectives, proposing to give new meaning to the way of thinking about and in academia^(4)^. In this way, it is presented in dialogue with the need for differentiated healthcare, mentioned in SASISUS, organized in Special Indigenous Health Districts (DSEI - *Distritos Sanitários Especiais Indígenas*) and in multidisciplinary teams, which work based on interculturality^(5)^, and which have, in its composition, nurses and indigenous health workers.

There are, however, challenges for indigenous students in higher education that include linguistic, geographic and socioeconomic barriers^(2,4,5)^, which need to be better explored in the literature. Therefore, the discussion of these limitations in training is essential, considering that this issue goes beyond the Quota Law and goes beyond human rights and equity in the SUS, especially in the context of the diversity of knowledge of professionals, aiming to reduce inequalities and inequities^(1)^.

Additionally, there is a lack of studies that address training and healthcare appropriate to communities and their different contexts^(5,6)^, essential to work at SASISUS and beyond. Likewise, there is little evidence on the ways in which indigenous and non-indigenous nurses are absorbed, based on selection processes and the implementation of the sociopolitical organization provided for in the Brazilian National Policy for Healthcare for Indigenous Peoples (PNASPI - *Política Nacional de Atenção à Saúde dos Povos Indígenas*)^(3)^. Therefore, this publication establishes coping with models^(3)^; guides ruptures with structures, which support prejudiced and discriminatory practices^(4)^; and presents paths^(7)^, pointing out strategies for including dialogues with the worldviews of indigenous peoples in the Brazilian health system^(6)^.

Furthermore, the text supports the differentiated approach, i.e., culturally sensitive to different indigenous peoples^(5,8)^, an aspect about which little has been discussed^(6)^ as well as offering subsidies to education and health policies, capable of implementing equitable flows, with significant implications for SASISUS and the entire SUS care network, in which these professionals work. Indigenous and non-indigenous nurses bring unique knowledge to the health network, and their inclusion at all levels of complexity in the SUS, from primary care to more specialized services, promotes healthcare that respects cultural diversity and ensures equity in access and quality of care^(6)^ beyond basic care, as they also work in other contexts, external to SASISUS.

This initiative is in line with the 2030 Agenda, as it seeks to establish, through goals 3 and 4 of the Sustainable Development Goals (SDGs), the necessary approach between education, health and human rights, establishing conditions to have mechanisms valuing diversity in nursing training^(6)^.

The problem question of this study focuses on achieving equity in higher education, more specifically on analyzing the implications of this condition on the SUS workforce^(9)^, justified by the indispensability of understanding its consequences at a global level^(1)^. For this relationship, a dialogue was created between students’ socio-historical processes, their potential and their implications, based on Lev Vygotsky’s theoretical model^(7)^, which deals with the interaction, relationships and transformations established with the environment to knowledge construction, which gives value to the historicity of these people, making it possible to point out indicators to eliminate disparities in education in different contexts^(1)^.

## OBJECTIVES

To analyze the possibilities and potential of training indigenous nurses, in relation to the SUS, understanding the relationships between education and health.

## METHODS

This is a theoretical-reflective study, aligned with the experience and critical thinking of its authors and supported by contemporary scientific literature and goals 3 and 4 of the SDGs, from the training of nurses in Brazil, with regard to health and quality education. A non-systematic search was carried out, in which six Brazilian studies were identified, carried out between 2010 and 2023, excluding theses, dissertations and studies that did not cover indigenous nurses as workers. These works were complemented by the authors’ on-site higher education experiences, as researchers and indigenous nurses in training, currently working at SASISUS, who shared their experiences in the training process, lending leading role to the study.

The text is structured into three thematic axes, resulting from the researchers’ reflective analysis of the topic, based on Lev Vygotsky’s theoretical model: Potentialities of including indigenous students in nursing training; Paths to achieving equity through inclusion and retention policies for indigenous students at different levels of education; and Implications of obtaining this condition for the Brazilian Health System and global health. The main points of discussion were indigenous students’ sociocultural universe and the potential of including the topic under discussion in university spaces and in knowledge production.

## DEVELOPMENT

### Potentials of including indigenous students in nursing training

The Brazilian Quota Law guarantees a percentage of places for indigenous students in federal higher education institutions, supporting the recognition of ethnic-racial diversity and culturally sensitive health practices adapted to different indigenous peoples, as provided for in the 1988 Federal Constitution. In the context of SASISUS, this issue has potentially significant implications, as it ensures that indigenous nurses are trained, contributing to the representation of this group in the profession for equal opportunities^(1,4)^ and quality of care, since, by sharing the culture and traditions of their patients, carry out equitable interventions^(1)^, with differentiated and decentralized healthcare, according to their specificities^(5)^.

This ratifies the right of indigenous peoples to difference, to their culture and to their social organization^(1)^, considering PNASPI, which provides for healthcare professionals’ work in an intercultural manner^(5,6)^. Likewise, the arrival at equanimity ratifies the principles indicated in the Federal Constitution of 1988 in SUS and SASISUS, as well as reflected in the Brazilian National Curricular Guidelines (DCN - *Diretrizes Curriculares Nacionais*) for undergraduate nursing courses (Resolution CNE/CES 3/2001), aiming to achieve equity. They seek to predict the population’s health needs, comprehensive care and critical and reflective training, also guaranteed by DCN for indigenous school education (Resolution CNE/CEB 3/1999), which contributes to achieving equity in training of professionals based on diversity of knowledge.

Although the practices in question are aimed at basic education, they establish the principle of interculturality. Furthermore, Law 12,288 of July 2, 2010, which established the Statute of Racial Equality, seeks to guarantee equal opportunities and combat racial discrimination^(4)^, aiming to reduce social inequities and poverty among marginalized groups^(1)^.

Furthermore, there are several important potentialities in the adoption of indigenous knowledge and medicines in terms of a respectful dialogue with biomedicine^(2,4,5)^. Such an attitude can contribute to debates about the inclusion of other epistemologies in the concepts and practices of healthcare, not only in the field of training indigenous people, but in the training of non-indigenous people and the intercultural qualification of teaching staff^(6)^. Likewise, this knowledge enriches training, through case studies, medicalization processes, health models^(3)^, indigenous cultures and traditions, enabling a perspective on the way in which they relate to health and communications intercultural and linguistic^(5,6)^, within a transformative worldview^(7)^.

Despite the opportunities created, there are challenges associated with communication^(6)^, because the mother tongue of a significant part of Brazilian indigenous people and its different peoples is not Portuguese. For this reason, students and academics have mandatory contact and need to master Portuguese, even as a second language. This condition is also identified as a barrier in the path of healthcare for indigenous peoples in the SUS^(6)^. There is a need, therefore, for a fruitful debate about the limitations in training and use of interpreters in care processes to achieve equity between health and education^(1)^.

To achieve this, the academic universe must shed light on learning as a social and cultural process, not just an individual one, as indigenous students have the potential to learn, interacting with other students, with different potentials. In the context of higher education, this may mean that professors and other students play a crucial role in supporting indigenous students to reach their maximum potential, with interaction environment being the ideal promoter^(7)^.

Regarding cultural and socioeconomic issues of a historical nature, there are limitations related to marginalization of this group in society, the quality of basic education^(4)^ and the deficit in the use of and access to quality technologies that support learning methods. When indigenous students are not understood, this highlights prejudices and lack of knowledge, based on historical and sociopolitical issues and conditions of social inequality^(9)^, which includes them as beneficiaries of affirmative actions^(1,2,4)^.

When incorporating this model, it is necessary to integrate interculturality into education to understand the reality of indigenous territories^(9)^, dialoguing and training to obtain knowledge about languages and cultural values of indigenous patients relevant to the absorption of cultural nuances present in communication. In this regard, it is important to encourage cultural self-awareness, in order to reflect students’ and non-indigenous students’ conduct as a whole, specifically, aiming to understand how they can influence patient care and how they can be trained to defend their communities, working to eliminate disparities and ensure equitable access to healthcare^(6,8)^.

It should be noted that this aspect still contributes to fighting for the rights of indigenous peoples, preparing them to take on leadership roles, for instance, in a global perspective^(1)^, considering the peculiarities of each ethnic group, with a focus on health promotion and prevention of emerging diseases, such as malaria, leishmaniasis, tuberculosis and their diseases, such as violence present in indigenous territories^(8)^. There are several possibilities for training in care coordination and interdisciplinary teamwork, establishing elements for integrated health practices^(5)^, in line with work with families, understanding their dynamics and needs^(7)^, which are commonly identified in DSEI, organizational part of SASISUS^(6)^.

### Paths to achieving equity through inclusion and retention policies for indigenous students at different levels of education

The starting point for achieving equity in education^(1)^ is the institutional awareness of professors, managers and administrative technicians about the importance of diversity and equity in the academic environment^(1,6)^ since graduation. Examples of this are monitoring and support, with system guarantees, with tutorials, mentoring, academic and psychological counseling, and other resources, to guarantee indigenous nursing students’ permanence, fundamental for developing human structures of thought and language^(7)^.

Curricular flexibility can recognize and value diverse knowledge and experiences, promoting an inclusive pedagogy that reflects the diversity of students^(10)^. Scholarship and aid programs reduce financial difficulties, whereas the promotion of inclusive environments, without discrimination, prejudice or harassment, can be carried out through events, seminars and discussions on diversity, inclusion and equity.

Students’ active participation in discussions, decisions and policies concerning diversity and equity^(1,4)^ offers valuable insights into their experiences and needs^(6)^, generating potential to strengthen the relationship between the university and local communities, especially those from which indigenous students come. Initiatives such as student housing are also welcoming actions, whereas nursing education management can and should monitor indigenous students, in order to guarantee their access and permanence.

Regarding deficits in basic education, it is necessary to strengthen the transition from high school to higher education. This can occur through leveling and training programs, with strategies focused on inclusion and equity, which can be carried out by universities, both to admit and to support and attract students from various ethnicities, ensuring that they have the necessary tools and resources continued graduation as well as qualifications at other levels^(10)^.

One of the ways to reduce this deficit includes collaborative actions that promote active learning^(7)^. Indigenous students learn from the environment and those around them, benefiting from interactional pedagogical approaches with professor measurement that encourage higher psychological functions related to mental skills, social processes and the cultural and historical context^(7)^, which can be developed using adapted instruments and strategies, including the issue of language.

In relation to graduate studies, this level presents specific challenges, given that, in nursing, there is a reduced percentage of vacancies offered, including in regions with significant records of indigenous peoples^(10)^. Such access demands advances, given that permanence is incipient and needs to be reviewed, due to the characteristics of this level of training, since graduate studies have peculiar characteristics, such as knowledge production and length of dedication^(10)^. Therefore, it is essential that there is equitable selection and admission^(1)^, with transparent and inclusive selection processes, considering different experiences and even languages and procedures. An example of this is the adoption of special selection strategies, using commissions trained in inclusive processes, consisting of tests with different criteria, such as remote stages carried out from the territories, defense of a descriptive memorial, writing as a theme aligned with indigenous culture and oral test.

To provide this access, research groups need to offer training to indigenous students, from scientific initiation, characterized by inclusive processes, which guarantee identification of their needs, encouraging and supporting research that addresses diversity, equity and inclusion^(1,10)^. Furthermore, the importance of different perspectives in academic training and production must be recognized, with a notable impact on the healthcare of specific groups and the Brazilian population as a whole.

In terms of permanence, financial support in graduate studies must provide for an equitable distribution of places in such a way that students from underrepresented groups^(9)^ have priority in obtaining scholarships. This will be reflected in continued training, in coping with discrimination and access to quality technologies that promote a culture of equity^(1)^, benefiting graduate programs from the wealth of perspectives and experiences that a diverse community offers to the academic environment^(1,10)^.

Including indigenous nurses in graduate studies provides an opportunity to conduct research, resulting in innovations in nursing practice in indigenous contexts^(10)^. In the case of nurses with field experience, practical and relevant solutions can be formed on leadership, management, care and technologies^(8)^. Indigenous nurses taking doctoral and master’s degrees will contribute significantly to expanding and deepening the body of nursing knowledge, collaborating with professionals from other areas and promoting holistic and integrated views in training, equally^(5)^.

From this perspective, they can also contribute to formulating and assessing health policies, taking into account their practical experience and theoretical knowledge^(7,8)^, becoming professors and researchers and being involved in projects, from their perspective^(3)^. It is noteworthy that this enriches the academic environment, enhances research in health and solidifies nursing as a fundamental profession in health promotion and care in different contexts and levels^(10)^. Furthermore, it provides opportunities for international collaboration^(7)^, conferences and publications, raising the standard and recognition of Brazilian nursing on the world stage, including cooperating with global goals^(1)^.

### Implications of obtaining this condition for the Brazilian Health System and global health

Training indigenous nurses has implications for global health^(1,3,7,8)^, reverberating in different spheres: quality of direct care in communities; and in broader aspects of public policy and human rights^(1,3)^. For the SUS, training indigenous nurses allows for adequate care to the cultural reality^(5,7)^; initially, through Primary Healthcare (PHC), within DSEI, which enables a more effective result in health interventions and their different levels of complexity.

Considering the relationships between education and health^(1)^, when analyzing training for the SUS, one can see the expansion of views on healthcare services for indigenous peoples, contributing to universalization of health^(1)^. This also values the dynamics of people^(3)^, fundamental to the fight and prevention, identification and treatment of diseases and injuries, taking into account cultural specificities^(7)^.

In this way, bridges are built between the community and the health system, facilitating access to services and promoting community trust in the system^(2,7,8)^, due to the understanding of local dynamics and beliefs^(7,8)^. This fact therefore implies the effective screening and monitoring of diseases, reducing underreporting and changing health indicators^(1)^.

These processes can intervene in indicators such as mortality and morbidity rates, vaccination coverage, access to healthcare services, literacy rates and school completion, among others^(1)^. They therefore enhance the assessment of progress, regarding SDGs 3 and 4, providing quantitative assessments of health, well-being and quality education among indigenous communities and the population in general^(1)^.

Hence, it is a way to combat historical inequalities and promote health equity^(1)^, because, when working in their villages, indigenous nurses reinforce PHC, which is fundamental to disease prevention and health promotion^(1)^, given that they cooperate with appreciation and recognition of cultural diversity and traditional knowledge in combating endemic diseases^(1,2)^ in villages, observing their practices and therapeutic approaches. This results, after all, in assumptions of successful practices, presenting significant references to other countries that have indigenous peoples or cultural minorities^(1,9)^.

Likewise, the initiative provides opportunities for defending indigenous rights, a key aspect to reinforce the commitment to indigenous peoples’ rights on the international stage^(1,4)^, as well as aligning with SDGs, especially with regard to inclusive education, health and well-being and reducing inequalities^(1)^, in addition to representing a strategy that strengthens an equitable health system, which values cultural diversity on a global scale^(1,5)^.

To this end, curricula must consider the sociocultural reality^(5,7)^ and the specific challenges of training these groups for the SUS^(9)^, which can be established through solid partnerships between educational institutions and the service, facilitating clinical practice and integration of future nurses^(6-8)^ so that, instead of following exclusively biomedical models, this training dialogues with other knowledge, such as indigenous medicine^(3)^. This requires integrated public policies, partnerships between different sectors and a firm commitment to the principles of inclusion, respect and appreciation of indigenous medicine^(3,4)^.

This time, the importance of culture and social interaction^(7)^ in learning and health as promoters of more contextualized and culturally relevant training for indigenous and non-indigenous nurses^(6,7)^, promoting more inclusive and effective health and education among indigenous peoples, with repercussions in different layers of the SUS and of global health^(1)^.

## FINAL CONSIDERATIONS

This reflection sought to highlight the possibilities of equitable training of indigenous nurses for SUS, understanding how established relationships are opportune to expand healthcare possibilities beyond the Brazilian context. As a workforce for public health, training indigenous nurses is of great relevance in SASISUS, in the same way as in the care network, which considers the peculiarities and diversities highlighted in legislation and in DCN for undergraduate nursing, which brings opportunities to listen and implement a new perspective in the face of emerging challenges.

The studies highlight the potential present in dialogue with elements of indigenous culture, as well as in debates about training and healthcare in multicultural contexts, through case studies that involve the diversities of indigenous peoples. Although inclusion and dialogue are deficient in the context of higher education, characterized by prejudices and racism, they can be addressed through the effective inclusion of content about indigenous peoples in curricula and selection processes from undergraduate to graduate access.

It is imperative to clarify that this study is limited to reflecting on training, and it is necessary to address the presence of different ethnicities at the performance level. It is recommended, however, to strengthen initiatives already implemented and expand access to other levels of training, such as master’s and doctoral degrees, with the intention of impacting research on training and care management, enabling the development of equitable products, in which indigenous students are leading actors. This condition ensures that knowledge production on the topic is inclusive and equitable, echoing in Brazilian regulatory frameworks and in the goals established by the United Nations regarding initiatives that add global health, such as climate issues and emerging diseases, established as a necessary condition for indigenous nurses to contribute to their own training.
