# Supplementary material for: Training of Brazilian indigenous nurses: between human rights, valuing diversity and inclusion
Source: Rev Bras Enferm. 2024 Dec 13;77(5):e20230430. doi: 10.1590/0034-7167-2023-0430 (PMC11654222; doi:10.1590/0034-7167-2023-0430)
Supplement: Translation

# Translation: Formação de enfermeiros e de enfermeiras indígenas brasileiros: entre direitos humanos, valorização da diversidade e inclusão

**DOI:** 10.1590/0034-7167-2023-0430pt

**Authors:** Nádile Juliane Costa de Castro, Ezequiel Sakew Wai Wai, Fernanda Teixeira Paes, Nyvia Cristina dos Santos Lima

**Affiliations:** I Universidade Federal do Pará. Belém, Pará, Brasil

**Keywords:** Enfermeiros e Enfermeiras, Povos Indígenas, Direitos Humanos, Diversidade, Equidade e Inclusão, Desenvolvimento Sustentável

## Abstract

**Objetivos:**

analisar as possibilidades e potencialidades da formação de enfermeiras e enfermeiros indígenas, diante do Sistema Único de Saúde (SUS), compreendendo as relações entre educação e saúde.

**Métodos:**

estudo teórico-reflexivo, fundamentado na literatura científica, alinhado à experiência, pensamento crítico de seus autores e às metas dos Objetivos de Desenvolvimento Sustentável no Brasil.

**Resultados:**

esse texto articula três eixos: Potencialidades de inclusão de estudantes indígenas na formação em enfermagem; Caminhos para a efetivação da equidade por meio das políticas de inclusão e permanência de estudantes indígenas em diferentes níveis; e Implicações disto para o SUS e a saúde global.

**Considerações Finais:**

estudantes indígenas, beneficiários de ações afirmativas, enfrentam desafios de inclusão e permanência nas universidades públicas que impactam diretamente a formação acadêmica. Somam-se a isto as dificuldades identificadas na educação básica, na formação docente e na implementação de políticas de permanência, com reflexos na atuação nos serviços e formação em outros níveis.

## INTRODUÇÃO

No Brasil, há 1,6 milhão de indígenas com direitos previstos na Constituição Federal de 1988 e em tratados internacionais, entre eles estudantes indígenas, para quem o acesso à educação superior é um direito necessário para diminuir iniquidades sociais e em saúde^(1)^. A formação de indígenas como acadêmicos em cursos da área da saúde, de que trata este estudo, reafirma o princípio da equidade, sinalizado no Sistema Único de Saúde (SUS) e no Subsistema de Atenção à Saúde Indígena do SUS (SASISUS), pensado a partir das singularidades dos diferentes povos indígenas. Reconhece-se, aqui, a importância da participação de indígenas e de suas visões de saúde e de bem-estar^(2,3)^, fundamentais para dialogar e enfrentar as iniquidades presentes nos diferentes contextos de saúde^(1,2)^.

Estudos neste sentido trazem à tona debates sobre processos e estruturas equânimes, dialogando sobre o caráter pluriétnico da população brasileira^(3)^. Tratam, também, dos potenciais impactos de políticas públicas no Brasil, como a Lei de Cotas, formalizada na Lei Federal nº 12.711, de 29 de agosto de 2012, atualizada pela Lei nº 14.723/2023, que assegura que indígenas tenham oportunidade de acesso ao ensino superior por meio de perspectivas cidadã e de diversidade, incorporadas a todos os níveis^(1,4)^.

Essa política reconhece a diversidade étnico-racial e resguarda modos de ser e de estar de povos indígenas^(3,4)^, sendo importante para a formação intercultural de todos os profissionais de saúde que dela participam, provocando novas dinâmicas nas universidades, viabilizando diálogos e interações entre diferentes culturas e produções de conhecimentos, sob as perspectivas interdisciplinar e de diversidade, propondo ressignificar o modo de pensar a e na academia^(4)^. Desse modo, apresenta-se em diálogo com a necessidade de atenção diferenciada em saúde, citada no SASISUS, organizado em Distritos Sanitários Especiais Indígenas (DSEI) e em equipes multidisciplinares, que atuam a partir da interculturalidade^(5)^, e que têm, em sua composição, enfermeiros e agentes de saúde indígenas.

Há, no entanto, desafios aos estudantes indígenas na educação superior que incluem questões linguísticas, geográficas e socioeconômicas^(2,4,5)^, que precisam ser mais bem exploradas na literatura. Portanto, a discussão dessas limitações na formação é imprescindível, observado que tal questão vai além da Lei de Cotas e perpassa os direitos humanos e a equidade no SUS, sobretudo no contexto da diversidade de saberes dos profissionais, visando à diminuição de desigualdades e de iniquidades^(1)^.

Adicionalmente, há carência de estudos que abordem formação e cuidados de saúde apropriados a comunidades e seus diferentes contextos^(5,6)^, essenciais à atuação no SASISUS e além deste. Do mesmo modo, há poucas evidências sobre as formas de absorção de enfermeiros indígenas e não indígenas, a partir dos processos seletivos e da implementação da organização sociopolítica prevista na Política Nacional de Atenção à Saúde dos Povos Indígenas (PNASPI)^(3)^. Portanto, essa publicação estabelece o enfrentamento de modelos^(3)^; norteia rupturas com estruturas, que corroboram práticas preconceituosas e discriminatórias^(4)^; e apresenta caminhos^(7)^, apontando estratégias à inclusão de diálogos com as cosmovisões de povos indígenas no sistema brasileiro de saúde^(6)^.

Além disso, o texto corrobora a abordagem diferenciada, ou seja, culturalmente sensível aos diferentes povos indígenas^(5,8)^, aspecto sobre o qual pouco se tem dialogado^(6)^, assim como oferece subsídios a políticas de educação e de saúde, capazes de implementar fluxos equânimes, com implicações significativas no SASISUS e em toda a rede de atenção do SUS, em que esses profissionais atuam. Enfermeiras e enfermeiros indígenas e não indígenas trazem conhecimentos únicos para a rede de saúde, e sua inclusão em todos os níveis de complexidade do SUS, desde a atenção primária até os serviços mais especializados, promove uma assistência à saúde que respeita a diversidade cultural e assegura a equidade no acesso e na qualidade do cuidado^(6)^ para além da atenção básica, em virtude de atuarem também em outros contextos, externos ao SASISUS.

Tal iniciativa vai ao encontro da Agenda 2030, uma vez que busca assentar, por meio das metas 3 e 4 dos Objetivos de Desenvolvimento Sustentável (ODS), as aproximações necessárias entre educação, saúde e direitos humanos, assim como estabelece condições para dispor de mecanismos de valorização da diversidade na formação de enfermeiras e enfermeiros^(6)^.

A questão problema deste estudo se concentra na obtenção da equidade na formação superior, mais especificamente na análise das implicações dessa condição na força de trabalho do SUS^(9)^, justificando-se pela imprescindibilidade de compreender seus desdobramentos em nível global^(1)^. Para essa relação, elaborou-se um diálogo entre processos sócio-históricos de estudantes, suas potencialidades e suas implicações, a partir do modelo teórico de Lev Vygotsky^(7)^, que trata da interação, das relações e das transformações estabelecidas com o meio para a construção do conhecimento, que confere valor à historicidade desses povos, assim como possibilita apontar indicadores para eliminar disparidades na educação em diferentes contextos^(1)^.

## OBJETIVOS

Analisar as possibilidades e potencialidades da formação de enfermeiras e enfermeiros indígenas, diante do SUS, compreendendo as relações entre educação e saúde.

## MÉTODOS

Trata-se de estudo teórico-reflexivo, alinhado à experiência e ao pensamento crítico de seus autores e sustentado na literatura científica contemporânea e nas metas 3 e 4 dos ODS, a partir da formação de enfermeiras e enfermeiros no Brasil, no que tange à saúde e à educação de qualidade. Foi realizada uma busca não sistemática, em que foram identificados seis estudos brasileiros, realizados entre 2010 e 2023, excluindo-se teses, dissertações e estudos que não abrangeram enfermeiros indígenas como trabalhadores. Esses trabalhos foram complementados pelas experiências em educação superior *in loco* dos autores, enquanto pesquisadores e enfermeiro indígena em formação, atualmente trabalhador do SASISUS, que compartilhou suas experiências no processo de formação, emprestando protagonismo ao estudo.

O texto está estruturado em três eixos temáticos, decorrentes da análise reflexiva dos pesquisadores sobre o tema, baseada no modelo teórico de Lev Vygotsky: Potencialidades da inclusão de estudantes indígenas na formação em enfermagem; Caminhos para efetivação da equidade por meio das políticas de inclusão e de permanência de estudantes indígenas em diferentes níveis de ensino; e Implicações da obtenção desta condição para o Sistema Único de Saúde e para a saúde global. Os principais pontos de discussão foram o universo sociocultural do estudante indígena e as potencialidades da inclusão do tema em comento nos espaços universitários e na produção de conhecimento.

## DESENVOLVIMENTO

### Potencialidades da inclusão de estudantes indígenas nas formações em enfermagem

A Lei de Cotas brasileira garante um porcentual de vagas a estudantes indígenas em instituições federais de ensino superior, corroborando o reconhecimento da diversidade étnico-racial e das práticas de saúde culturalmente sensíveis e adaptadas aos diferentes povos indígenas, como prevê a Constituição Federal de 1988. No contexto do SASISUS, essa questão tem implicações potencialmente significativas, pois assegura que sejam formados enfermeiros indígenas, contribuindo para a representatividade desse grupo na profissão para a igualdade de oportunidades^(1,4)^ e para a qualidade do atendimento, visto que, ao compartilharem a cultura e as tradições de seus pacientes, realizam intervenções equânimes^(1)^, com atenção diferenciada e descentralizada à saúde, de acordo com suas especificidades^(5)^.

Isto ratifica o direito dos povos indígenas à diferença, à sua cultura e à sua organização social^(1)^, considerando a PNASPI, que prevê a atuação de profissionais de saúde de maneira intercultural^(5,6)^. Igualmente, a chegada à equanimidade ratifica os princípios indicados na Constituição Federal de 1988 no SUS e no SASISUS, bem como reflete nas Diretrizes Curriculares Nacionais (DCN) para os cursos de graduação em enfermagem (Resolução CNE/CES nº 3/2001), visando ao alcance da equidade. Eles buscam prever as necessidades em saúde da população, a integralidade da assistência e as formações crítica e reflexiva, também garantidas pelas DCN para a educação escolar indígena (Resolução CNE/CEB nº 3/1999), o que colabora para o alcance da equidade na formação de profissionais, a partir da diversidade de saberes.

Embora as práticas em questão sejam voltadas à educação básica, estabelecem o princípio da interculturalidade. Além disso, a Lei nº 12.288, de 2 de julho de 2010, que instituiu o Estatuto da Igualdade Racial, procura garantir a igualdade de oportunidades e combater a discriminação racial^(4)^, objetivando diminuir iniquidades sociais e a pobreza de grupos marginalizados^(1)^.

Ademais, há diversas e importantes potencialidades na adoção de conhecimentos e de medicinas indígenas nos termos de um respeitoso diálogo com a biomedicina^(2,4,5)^. Tal atitude pode contribuir com os debates sobre inclusão de outras epistemologias nos conceitos e nas práticas sobre cuidados em saúde, não somente no campo da formação de indígenas, mas na formação de não indígenas e na qualificação intercultural do corpo docente^(6)^. Igualmente, esses conhecimentos enriquecem os treinamentos, por intermédio de estudos de caso, de processos de medicalização, de modelos de saúde^(3)^, de culturas e de tradições indígenas, viabilizando uma ótica sobre a forma pela qual se relacionam à saúde e às comunicações intercultural e linguística^(5,6)^, dentro de uma visão de mundo transformadora^(7)^.

Apesar das oportunidades criadas, existem desafios associados à comunicação^(6)^, porque a língua materna de parte significativa dos indígenas brasileiros e dos seus diferentes povos não é o português. Por essa razão, estudantes e acadêmicos têm contato obrigatório e precisam dominar o português, embora como sendo segunda língua. Essa condição é identificada como desafio também no percurso dos cuidados à saúde de povos indígenas no SUS^(6)^. Há necessidade, portanto, de um debate profícuo sobre as limitações na formação e sobre o emprego de intérpretes nos processos do cuidar para alcançar a equidade entre saúde e educação^(1)^.

Para isto, o universo acadêmico deve lançar luz sobre o aprendizado enquanto processo social e cultural, não apenas individual, na medida em que os estudantes indígenas têm potencial para aprender, interagindo com outros estudantes, com diferentes potenciais. No contexto do ensino superior, isso pode significar que os professores e os demais estudantes desempenham um papel crucial no apoio aos estudantes indígenas para alcançar seus potenciais máximos, sendo o ambiente de interação o promotor ideal^(7)^.

No que concerne às questões culturais e socioeconômicas de caráter histórico, há limitações relacionadas à marginalização deste grupo na sociedade, à qualidade da educação básica^(4)^ e ao déficit no uso e acesso a tecnologias de qualidade que subsidiem os métodos de aprendizagem. Quando estudantes indígenas não são compreendidos, isso evidencia preconceitos e desconhecimentos, amparados em questões históricas e sociopolíticas e em condições de desigualdade social^(9)^, o que os incluiu como beneficiários das ações afirmativas^(1,2,4)^.

Ao incorporar esse modelo, faz-se necessário integrar a interculturalidade à educação, para compreender a realidade dos territórios indígenas^(9)^, dialogando e capacitando para obter conhecimentos sobre línguas e valores culturais de pacientes indígenas relevantes à absorção das nuances culturais presentes na comunicação. Nesse sentido, é importante o incentivo à autoconsciência cultural, de modo a refletir as condutas dos estudantes como um tudo e dos estudantes não indígenas, especificamente, objetivando entender como esses podem influenciar o atendimento ao paciente e como podem ser treinados para defender suas comunidades, trabalhando para eliminar disparidades e garantir acesso equitativo nos cuidados em saúde^(6,8)^.

Ressalte-se que tal aspecto ainda contribui na luta por direitos de povos indígenas, preparando-os para assumir funções de liderança, por exemplo, em uma perspectiva global^(1)^, em vista das peculiaridades de cada etnia, com foco na promoção da saúde e na prevenção de doenças emergentes, como malária, leishmaniose, tuberculose e seus agravos, como as violências presentes nos territórios indígenas^(8)^. São múltiplas as possibilidades de treinamento em coordenações de cuidados e de trabalhos em equipe interdisciplinar, instituindo elementos para as práticas integradas de saúde^(5)^, consoantes com os trabalhos com famílias, compreendendo suas dinâmicas e suas necessidades^(7)^, as quais são comumente identificadas nos DSEI, parte organizacional do SASISUS^(6)^.

### Caminhos para efetivação da equidade por meio das políticas de inclusão e de permanência de estudantes indígenas em diferentes níveis de ensino

O ponto de partida para alcançar a equidade na educação^(1)^ é a conscientização institucional de docentes, gestores e técnicos-administrativos acerca da importância da diversidade e da equidade no ambiente acadêmico^(1,6)^ desde a graduação. Constituem exemplos disso o acompanhamento e o apoio, com garantias de sistemas, com tutorias, mentorias, aconselhamentos acadêmico e psicológico, e outros recursos, para garantir a permanência de estudantes de enfermagem indígenas, fundamentais para desenvolver estruturas humanas de pensamento e de linguagem^(7)^.

A flexibilização curricular pode reconhecer e valorizar saberes e experiências diversas, promovendo uma pedagogia inclusiva que reflita a diversidade do corpo estudantil^(10)^. Os programas de bolsas e de auxílios diminuem as dificuldades financeiras, enquanto que a promoção de ambientes inclusivos, sem discriminação, sem preconceito ou sem assédio, pode ser realizada a partir de eventos, seminários e discussões sobre diversidade, inclusão e equidade.

A participação ativa dos estudantes em discussões, decisões e políticas concernentes à diversidade e à equidade^(1,4)^ oferece *insights* valiosos sobre suas experiências e suas necessidades^(6)^, gerando potencial para fortalecer a relação entre a universidade e as comunidades locais, especialmente aquelas das quais os estudantes indígenas vêm. Iniciativas como moradias estudantis também são ações acolhedoras, ao passo que a gestão do ensino em enfermagem pode e deve acompanhar os estudantes indígenas, no intuito de garantir seu acesso e sua permanência.

No tocante aos déficits na educação básica, é preciso fortalecer a transição do ensino médio para o superior. Isso pode ocorrer por meio de programas de nivelamento e de capacitação, com estratégias focadas em inclusão e equidade, que podem ser realizadas pelas universidades, tanto para admitir quanto para apoiar e captar estudantes de várias etnias, garantindo que tenham as ferramentas e os recursos necessários à permanência na graduação, bem como à qualificação em outros níveis^(10)^.

Um dos modos de diminuir esse déficit inclui ações colaborativas que promovam a aprendizagem ativa^(7)^. O estudante indígena aprende com o meio e com aqueles que o circundam, beneficiando-se de abordagens pedagógicas interacionais com medição do educador que estimulem funções psicológicas superiores relativas às habilidades mentais, aos processos sociais e ao contexto cultural e histórico^(7)^, que podem ser desenvolvidas mediante instrumentos e estratégias adaptadas, incluindo a questão da linguagem.

Em relação à pós-graduação, tal nível apresenta desafios específicos, haja vista que, na enfermagem, há um porcentual reduzido de ofertas de vagas, inclusive em regiões com significativos registros de povos indígenas^(10)^. Tal acesso demanda avanços, dado que a permanência é incipiente e precisa ser revista, em virtude das características desse nível de formação, uma vez que a pós-graduação tem características peculiares, como a produção de conhecimento e o tempo de dedicação^(10)^. Portanto, é fundamental que haja seleção e admissão equitativas^(1)^, com processos seletivos transparentes e inclusivos, considerando experiências e, mesmo, línguas e procedimentos diversos. Um exemplo disso é a adoção de estratégias especiais de seleção, com o emprego de comissões treinadas em processos inclusivos, constituídos de provas com critérios diferenciados, como etapas remotas realizadas a partir dos territórios, defesa de memorial descritivo, redação como tema alinhado à cultura indígena e prova oral.

Para oportunizar esse acesso, os grupos de pesquisa precisam oferecer treinamentos a estudantes indígenas, desde a iniciação científica, caracterizados por processos inclusivos, que garantam identificação de suas necessidades, encorajando e apoiando pesquisas que abordem temas de diversidade, equidade e inclusão^(1,10)^. Além disso, deve-se reconhecer a importância de diferentes perspectivas na formação e produção acadêmica, com impacto notável na atenção à saúde de grupos específicos e da população brasileira como um todo.

No quesito permanência, o apoio financeiro na pós-graduação deve prever uma distribuição equitativa de vagas de tal maneira que estudantes de grupos sub-representados^(9)^ tenham prioridade na obtenção de bolsas. Isso se refletirá na formação continuada, no enfrentamento da discriminação e no acesso a tecnologias de qualidade que promovam uma cultura de equidade^(1)^, beneficiando os programas de pós-graduação a partir da riqueza de perspectivas e de experiências que uma comunidade diversificada oferece ao ambiente acadêmico^(1,10)^.

A inclusão de enfermeiros indígenas na pós-graduação oportuniza a condução de pesquisas, resultando em inovações na prática de enfermagem em contextos indígenas^(10)^. No caso de enfermeiros com experiência de campo, pode-se formar soluções práticas e relevantes sobre liderança, gestão, cuidados e tecnologias^(8)^. Enfermeiros indígenas doutorandos e mestrandos contribuirão significativamente para a expansão e o aprofundamento do corpo de conhecimentos da enfermagem, colaborando com profissionais de outras áreas e promovendo visões holística e integrada na formação, igualmente^(5)^.

Nessa perspectiva, eles também podem contribuir na formulação e avaliação de políticas de saúde, tendo em vista sua experiência prática e seu conhecimento teórico^(7,8)^, tornando-se professores e pesquisadores e se envolvendo em projetos, sob sua perspectiva^(3)^. Ressalta-se que isso enriquece o ambiente acadêmico, potencializa a pesquisa na área da saúde e solidifica a enfermagem como profissão basilar na promoção e no cuidado em saúde e em diferentes contextos e níveis^(10)^. Ademais, proporciona oportunidades de colaboração internacional^(7)^, de conferências e de publicações, elevando o padrão e o reconhecimento da enfermagem brasileira no cenário mundial, inclusive cooperando com metas globais^(1)^.

### Implicações da obtenção desta condição para o Sistema Único de Saúde e para a saúde global

A formação de enfermeiros indígenas traz implicações para a saúde global^(1,3,7,8)^, reverberando em diferentes esferas: na qualidade do atendimento direto nas comunidades; e nos aspectos mais amplos de políticas públicas e de direitos humanos^(1,3)^. Para o SUS, a formação de enfermeiros indígenas permite um atendimento adequado à realidade cultural^(5,7)^; inicialmente, pela Atenção Primária à Saúde (APS), dentro dos DSEI, o que possibilita um resultado mais eficaz nas intervenções em saúde e nos seus diversos níveis de complexidade.

Considerando as relações entre educação e saúde^(1)^, ao analisar a formação para o SUS, percebe-se a ampliação de olhares sobre os serviços de saúde para os povos indígenas, contribuindo para a universalização da saúde^(1)^. Isso também valoriza as dinâmicas dos povos^(3)^, fundamentais ao combate e à prevenção, à identificação e ao tratamento de doenças e de agravos, levando em conta as especificidades culturais^(7)^.

Desse modo, constroem-se pontes entre a comunidade e o sistema de saúde, facilitando o acesso a serviços e promovendo a confiança da comunidade no sistema^(2,7,8)^, em virtude da compreensão das dinâmicas e das crenças locais^(7,8)^. Tal fato implica, portanto, o rastreamento e o acompanhamento efetivo de agravos, diminuindo subnotificações e mudando indicadores de saúde^(1)^.

Esses processos podem intervir em indicadores, como taxas de mortalidade e de morbidade, coberturas de vacinação, acessos a serviços de saúde, taxas de alfabetização e conclusão escolar, entre outros^(1)^. Eles potencializam, portanto, a avaliação do progresso, concernente às metas dos ODS 3 e 4, proporcionando avaliações quantitativas da saúde, do bem-estar e da educação de qualidade, junto às comunidades indígenas e à população em geral^(1)^.

Nesse sentido, trata-se de uma forma de combater desigualdades históricas e promover a equidade em saúde^(1)^, pois, ao atuarem em suas aldeias, enfermeiros indígenas reforçam a APS, fundamental à prevenção das doenças e à promoção da saúde^(1)^, haja vista que cooperam com a valorização e o reconhecimento da diversidade cultural e dos saberes tradicionais no enfrentamento de endemias^(1,2)^ nas aldeias, observando suas práticas e suas abordagens terapêuticas. Isso resulta, afinal, em pressupostos de práticas exitosas, apresentando referências significativas a outros países que possuem povos indígenas ou minorias culturais^(1,9)^.

Igualmente, a iniciativa oportuniza a defesa de direitos indígenas, aspecto primordial para reforçar o compromisso com os direitos dos povos indígenas no cenário internacional^(1,4)^, assim como se alinha aos ODS, sobretudo no que tange à educação inclusiva, à saúde e ao bem-estar e à redução das desigualdades^(1)^, além de representar uma estratégia que fortalece um sistema de saúde equânime, o qual valoriza a diversidade cultural em escala global^(1,5)^.

Para tanto, os currículos devem considerar a realidade sociocultural^(5,7)^ e os desafios específicos de formação desses grupos para o SUS^(9)^, os quais podem ser instaurados por parcerias sólidas entre as instituições de ensino e o serviço, facilitando a prática clínica e a integração dos futuros enfermeiros^(6-8)^, de forma que, em vez de seguir modelos exclusivamente biomédicos, essa formação dialogue com outros conhecimentos, como os das medicinas indígenas^(3)^. Isso exige políticas públicas integradas, parcerias entre diversos setores e um compromisso firme com os princípios de inclusão, de respeito e de valorização das medicinas indígenas^(3,4)^.

Desta feita, verifica-se a importância da cultura e da interação social^(7)^ no aprendizado e na saúde como promotores de uma formação mais contextualizada e culturalmente mais relevante para enfermeiros^(6,7)^ indígenas e não indígenas, promovendo saúde e educação mais inclusivas e mais eficazes entre os povos indígenas, com reflexos em diversas camadas do SUS e da saúde global^(1)^.

## CONSIDERAÇÕES FINAIS

Esta reflexão buscou evidenciar as possibilidades da formação equânime de enfermeiros indígenas para o SUS, compreendendo como as relações estabelecidas são oportunas para ampliar possibilidades de cuidado em saúde para além do contexto brasileiro. Como força de trabalho para a saúde pública, a formação de enfermeiros indígenas detém grande relevância no SASISUS, do mesmo modo que na rede de atenção, que considera as peculiaridades e as diversidades apontadas nas legislações e nas DCN de graduação em enfermagem, o que traz as oportunidades de ouvir e de implementar uma nova perspectiva diante dos desafios emergentes.

Os estudos evidenciam as potencialidades presentes no diálogo com elementos da cultura indígena, bem como nos debates sobre treinamentos e sobre cuidados em saúde em contextos multiculturais, através de estudos de casos que envolvam as diversidades dos povos indígenas. Embora a inclusão e o diálogo sejam deficitários no contexto da educação superior, caracterizado por preconceitos e por racismo, os mesmos podem ser enfrentados por meio de inclusão efetiva de conteúdos sobre povos indígenas nos currículos e nos processos seletivos desde a graduação até o acesso desses à pós-graduação.

Imperioso elucidar que este estudo se limita a refletir sobre a formação, sendo necessário abordar a presença das diferentes etnias no nível da atuação. Recomenda-se, contudo, fortalecer iniciativas já implantadas e ampliar acessos a outros níveis de formação, como o mestrado e o doutorado, com o intento de impactar pesquisas sobre formação e gestão de cuidados, viabilizando o desenvolvimento de produtos equânimes, nos quais os estudantes indígenas sejam protagonistas. Tal condição assegura que a produção de conhecimento sobre o tema seja inclusiva e equânime, ecoando nos marcos regulatórios brasileiros e nas metas estabelecidas pela Organização das Nações Unidas quanto a iniciativas que agreguem a saúde global, como o caso das questões climáticas e dos agravos emergentes, estabelecidas como condição necessária para que enfermeiros indígenas contribuam para a própria formação.
